# Effect of novel inhaler technique reminder labels on the retention of inhaler technique skills in asthma: a single-blind randomized controlled trial

**DOI:** 10.1038/s41533-017-0011-4

**Published:** 2017-02-09

**Authors:** Iman A. Basheti, Nathir M. Obeidat, Helen K. Reddel

**Affiliations:** 1 Department of Clinical Pharmacy & Therapeutics, Faculty of Pharmacy, Applied Science Private University, Amman, Jordan; 2 Department of Internal Medicine, Faculty of Medicine, University of Jordan, Respiratory and sleep Medicine, Jordan University Hospital, Amman, Jordan; 3 Woolcock Institute of Medical Research, University of Sydney, Camperdown, NSW Australia

## Abstract

Inhaler technique can be corrected with training, but skills drop off quickly without repeated training. The aim of our study was to explore the effect of novel inhaler technique labels on the retention of correct inhaler technique. In this single-blind randomized parallel-group active-controlled study, clinical pharmacists enrolled asthma patients using controller medication by Accuhaler [Diskus] or Turbuhaler. Inhaler technique was assessed using published checklists (score 0–9). Symptom control was assessed by asthma control test. Patients were randomized into active (ACCa; THa) and control (ACCc; THc) groups. All patients received a “Show-and-Tell” inhaler technique counseling service. Active patients also received inhaler labels highlighting their initial errors. Baseline data were available for 95 patients, 68% females, mean age 44.9 (SD 15.2) years. Mean inhaler scores were ACCa:5.3 ± 1.0; THa:4.7 ± 0.9, ACCc:5.5 ± 1.1; THc:4.2 ± 1.0. Asthma was poorly controlled (mean ACT scores ACCa:13.9 ± 4.3; THa:12.1 ± 3.9; ACCc:12.7 ± 3.3; THc:14.3 ± 3.7). After training, all patients had correct technique (score 9/9). After 3 months, there was significantly less decline in inhaler technique scores for active than control groups (mean difference: Accuhaler −1.04 (95% confidence interval −1.92, −0.16, *P* = 0.022); Turbuhaler −1.61 (−2.63, −0.59, *P* = 0.003). Symptom control improved significantly, with no significant difference between active and control patients, but active patients used less reliever medication (active 2.19 (SD 1.78) vs. control 3.42 (1.83) puffs/day, *P* = 0.002). After inhaler training, novel inhaler technique labels improve retention of correct inhaler technique skills with dry powder inhalers. Inhaler technique labels represent a simple, scalable intervention that has the potential to extend the benefit of inhaler training on asthma outcomes.

## Asthma: Reminder labels improve inhaler technique

Personalized labels on asthma inhalers remind patients of correct technique and help improve symptoms over time. Iman Basheti at the Applied Science Private University in Jordan and co-workers trialed the approach of placing patient-specific reminder labels on dry-powder asthma inhalers to improve long-term technique. Poor asthma control is often exacerbated by patients making mistakes when using their inhalers. During the trial, 95 patients received inhaler training before being split into two groups: the control group received no further help, while the other group received individualized labels on their inhalers reminding them of their initial errors. After three months, 67% of patients with reminder labels retained correct technique compared to only 12% of controls. They also required less reliever medication and reported improved symptoms. This represents a simple, cheap way of tackling inhaler technique errors.

## Introduction

Asthma is a chronic inflammatory disease of the airways affecting millions worldwide.^1^ In developing countries, asthma is common, with relatively high prevalence amongst indigenous and urbanized people,^2, 3^ and the prevalence of asthma has been increasing.^4^ Medications used in the management of asthma are most effectively delivered by the inhalation route, with pressurized metered dose inhaler (pMDIs) and dry powder inhalers (DPIs) being the most frequently used.^1, 5–7^ DPIs are becoming more popular, as they avoid the co-ordination problems commonly associated with pMDIs.^8^

However, patients using DPIs still need to carry out a set of steps correctly to obtain optimal drug delivery, including preparing the device for inhalation and generating an inspiratory flow rate of at least 30L/min.^9^ Only 7–46% of Turbuhaler (TH) users^10^ and 13–50% of Accuhaler (ACC [Diskus] users)^10^ are found to have correct technique, and incorrect technique is associated with worse asthma outcomes.^11^ Training to improve the inhaler technique should thus be an important component of asthma education. Verbal instruction, physical demonstration, and written information are all important.^12^

With training, most patients can achieve correct inhaler technique, but this falls away rapidly within a few weeks or months.^13–16^ Several explanations have been proposed: patients forget with time; some inhaler devices are more difficult to use;^8^ some patients simply revert rapidly to their long-standing habits after education;^17^ and some patients with chronic conditions may choose not to follow medical advice.^18^ Strategies are needed in order to prevent this decline in the inhaler technique.

We have previously shown, in a 6-month cluster randomized controlled trial, that a brief educational service about inhaler technique was highly effective in improving inhaler technique and asthma outcomes for patients using ACC or TH.^19^ This service included an inhaler label on which the patient’s incorrect steps were highlighted on the inhaler technique checklist, to provide daily education between dispensing visits. However, the individual contribution of the labels themselves has not previously been assessed.

**Fig. 1:**
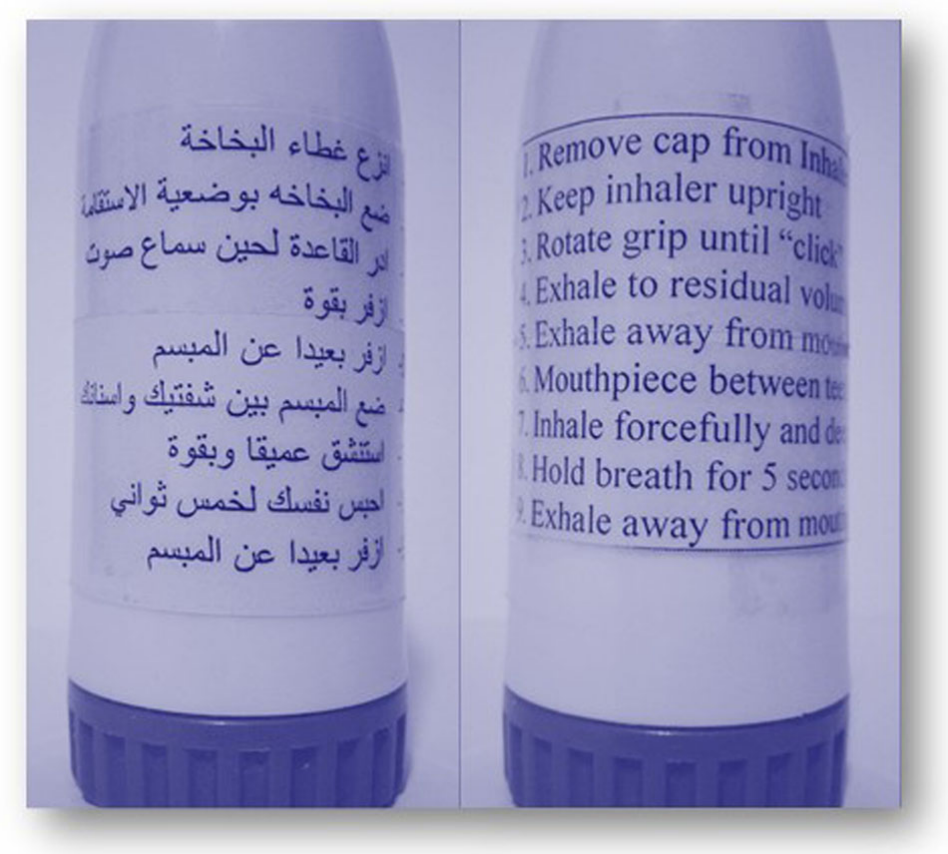
Novel Inhaler Technique Labels. At the baseline visit, the label was highlighted with any step, which the patient performed incorrectly at the initial assessment. The labels were printed in the Arabic language as shown in the first photo

The aim of the present study was to examine the effect of inhaler technique labels on retention of correct inhaler technique and on asthma control, following inhaler training, in patients with asthma. (Fig. 1).

## Results

### Patient characteristics

**Fig. 2:**
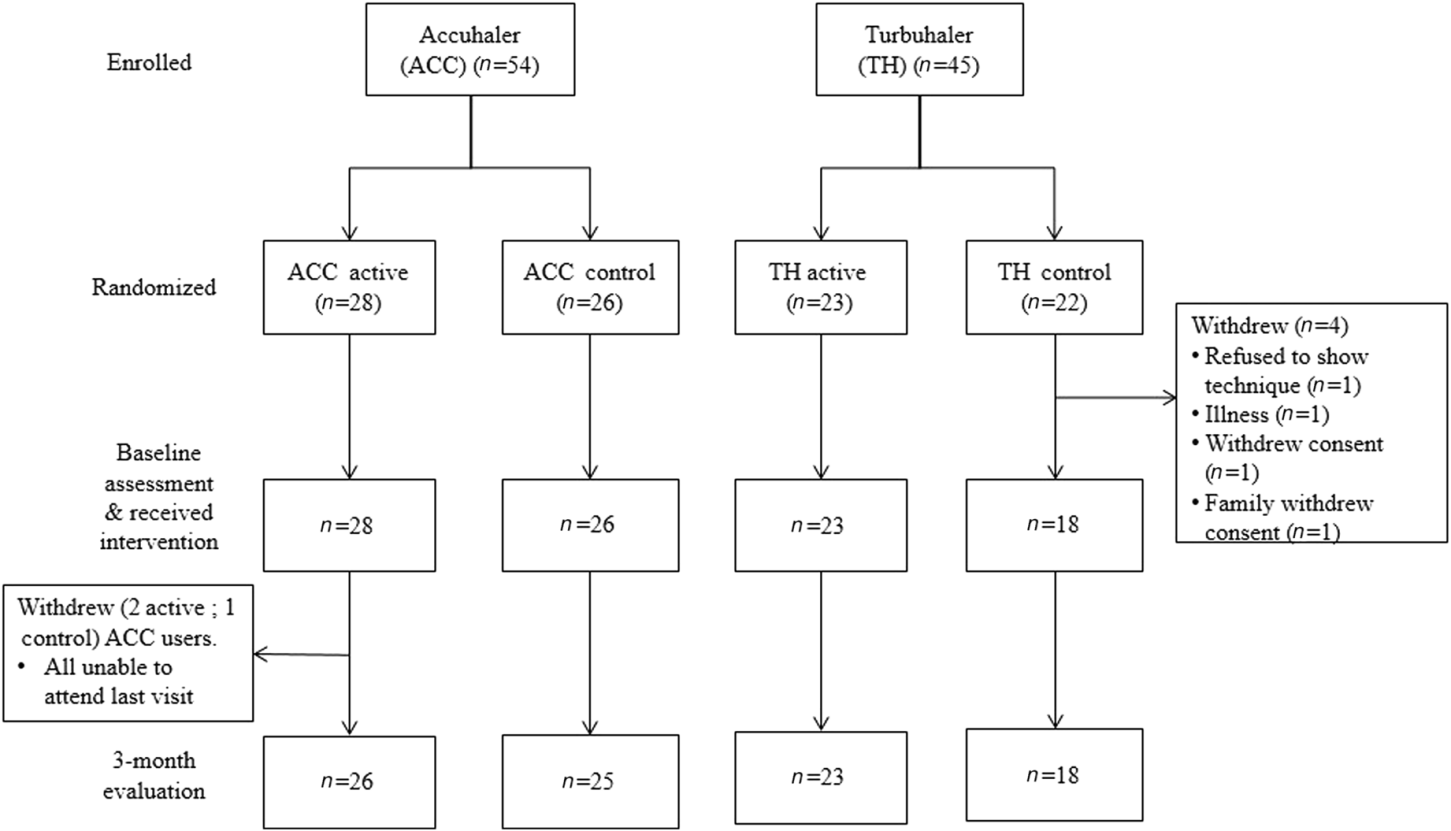
Flow chart of study participation. For the four patients who withdraw prior to baseline inhaler assessment (two males, two females), there were no significant differences from remaining patients in mean age (44 (SD 5.0 years, *P* = 0.84) or mean ACT score (15.75 (SD 2.22, *P* = 0.184)

Ninety-nine patients were enrolled into the study and randomised (54 ACC, 45 TH, Fig. 2). The mean age of participants was 44.9 (SD 15.2) years, and the majority were female (68%). No clinically important differences were seen in demographic or baseline characteristics between the randomization groups (Table 1). At baseline, mean ACT score was 13.1 (SD 4.2) for active group patients and 13.4 (SD 3.6) for control group patients, with only 5/99 (5%) participants having well-controlled asthma (ACT score >19). Baseline reliever use was also consistent with poorly controlled asthma (mean active 5.5 (SD 2.2), control 5.4 (2.3) puffs/day) (Table 1).

**Table 1:** Baseline demographics and characteristics for ACC (*n* = 54), TH (*n* = 45) users in the active and control groups

	ACC		TH		All	
Variable	Active *n* = 28	Control *n* = 26	Active *n* = 23	Control^a^ *n* = 22	Active *n* = 51	Control *n* = 48
Age, mean (SD)	45.3 (15.0)	49.4 (17.3)	40.8 (13.8)	43.2 (13.8)	43.25 (14.5)	46.6 (15.9)
Gender, females, *n* (%)	15 (54 %)	21 (81%)	16 (70%)	15 (68%)	31 (61%)	36 (75%)
Patient working, yes, *n* (%)	16 (57%)	10 (38%)	10 (43%)	8 (36%)	26 (51%)	17 (35%)
Amman location^b^, *n* (%) East:West:Outside	9:11:8 32:39:29	10:9:7 (38%:35%:27%﻿)	8:7:8 (35%:30%:35%)	8:9:5 (36%:41%:23%)	17:18:16 (33%:35%:31%)	18:18:12 (38%:37%:25%)
Smoking status, *n* (%) current: non-smoker: ex-smoker	4:21:3 (14%:75%:11%)	4:21:1 (15%:81%:4%)	1:20:2 (4%:87%:9%)	0:20:2 (0%:91%:9%)	5:41:5 (10%:80%10%)	4:41:3 (8%:85%:7%)
Age of onset of asthma, *n* (%) Infant:2–12:>12y	0:4:24 0%:14%:86%	0:2:24 (0%:8%:92%)	2:2:19 (9%:9%:82%)	1:4:17 (5%:14%:82%)	2:6:43 (4%:12%:84%)	1:6:42 (2%:11%:88%)
Duration of preventer use, years (SD)	8.1 (11.9)	9.7 (11.0)	8.8 (7.8)	9.4 (8.9)	8.5 (9.8)	9.5 (9.7)
Reliever use, puffs/day, mean (SD)	4.9 (2.4)	5.0 (2.5)	6.3 (1.8)	6.0 (1.9)	5.5 (2.2)	5.4 (2.3)
ACT score^c^, mean (SD)	13.9 (4.3)	12.7 (3.3)	12.1 (3.9)	14.3 (3.7)	13.1 (4.2)	13.4 (3.6)
Inhaler technique score, mean (SD)	5.3 (1.0)	5.5 (1.1)	4.7 (0.9)	4.2 (1.0)	5.0 (1.0)	5.0 (1.2)

^a^ Four TH users randomized into the control group withdrew from the study for different reasons, before baseline inhaler technique assessment (see details in Fig. 2).

^b^ East Amman (lower socioeconomic areas); West Amman (higher socioeconomic areas); outside Amman (mixed socioeconomic areas).

^c^ *ACT* asthma control test (score 5–25, higher indicates better asthma symptom control in the previous 4 weeks).

Four participants withdrew from the study after randomization and prior to baseline inhaler technique assessment; all were in the TH control group (Fig. 2, Table 1). A modified intention-to-treat analysis was therefore performed, based on the 95 participants with baseline inhaler technique scores.

### Baseline inhaler technique pre and post-education

**Fig. 3:**
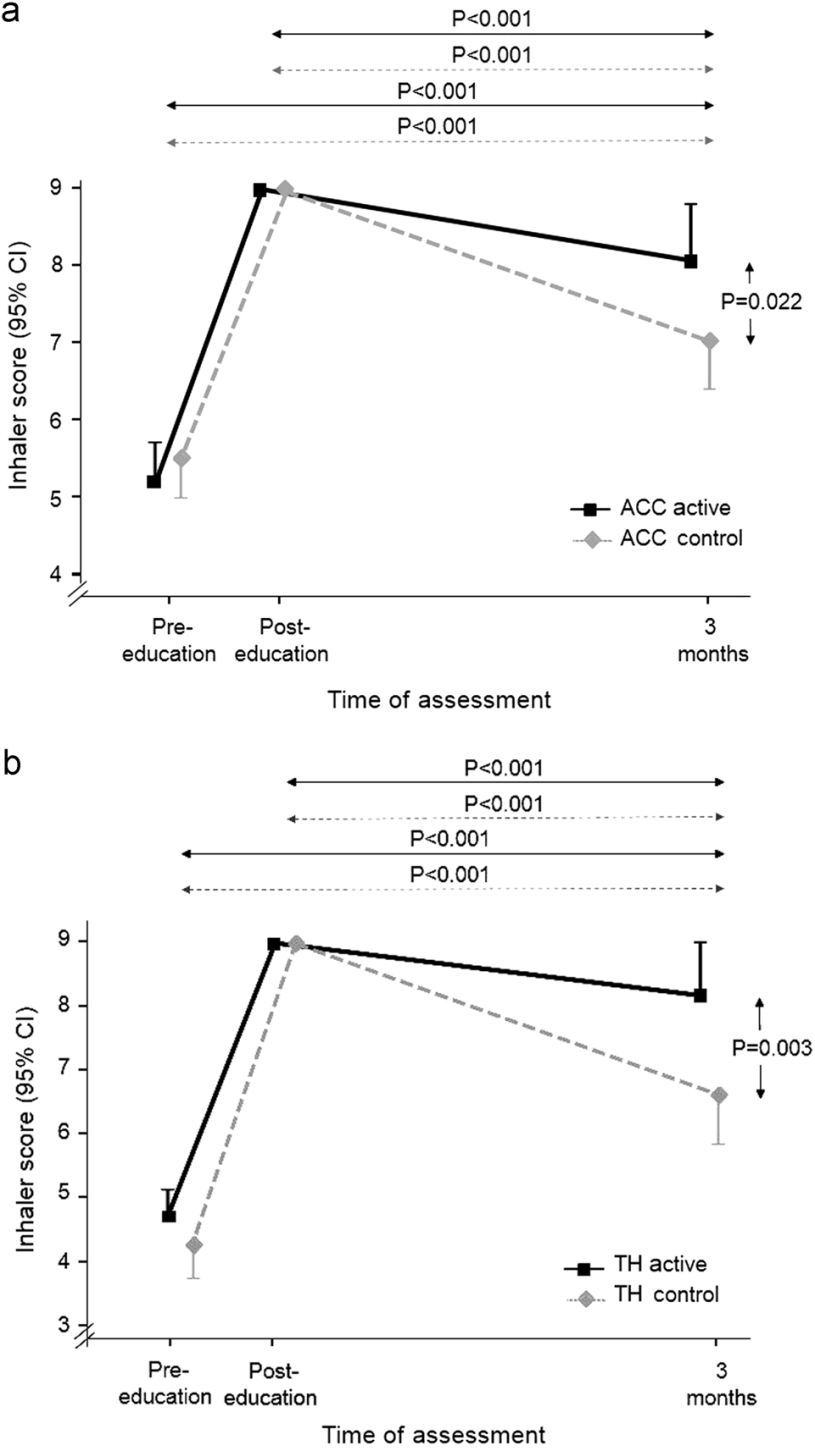
Mean inhaler technique score. **a** ACC users in the active (*n* = 28) and control (*n* = 26) groups. **b** TH users in the active (*n* = 23) and control (*n* = 18) groups

At baseline, pre-education, inhaler technique scores were low for all groups (Fig. 3a,b). The majority of ACC users demonstrated correct essential technique (active: 68%, control: 85%); however, few TH users demonstrated correct essential technique (active: 4%, control 0%) (Table 2). Only 1/95 (1%) patients (an ACC user) demonstrated all steps correctly. After education, all patients demonstrated all steps correctly (score 9/9, change in score from pre-education *P* < 0.001 for all, Table 2).

**Table 2:** Proportion of patients with correct technique (all steps correct) and correct essential technique (essential steps correct), for ACC (*n* = 54) and TH (*n* = 41) users in the active and control groups

A. Correct inhaler technique (i.e. score 9/9), *n* (%)				
ACC	Time of assessment	Active group (*n* = 28)	Control group (*n* = 26)	*P* value
	Baseline pre-education	0 (0%)	1 (4%)	0.295
	Baseline post-education	28 (100%)	26 (100%)	NA
	3 months^a^	16 (62%)	3 (12%)	<0.001
TH	Time of assessment	Active group (*n* = 23)	Control group (*n* = 18)	*P* value
	Baseline pre-education	0 (0%)	0 (0%)	NA
	Baseline post-education	23 (100%)	18 (100%)	NA
	3 months	17 (74%)	2 (11%)	<0.001

B. Correct essential technique, *n* (%)				
ACC	Time of assessment	Active group (*n* = 28)	Control group (*n* = 26)	*P* value
	Baseline pre-education	19 (68%)	22 (85%)	0.150
	Baseline post-education	28 (100%)	26 (100%)	NA
	3 months^a^	23 (88%)	21 (84%)	0.643
TH	Time of assessment	Active group (*n* = 23)	Control group (*n* = 18)	*P* value
	Baseline pre-education	1 (4%)	0 (0%)	0.370
	Baseline post-education	23 (100%)	18 (100%)	NA
	3 months	19 (83%)	3 (17%)	<0.001

For ‘essential’ steps for each device, see Supplementary online appendix. The only difference between active and control interventions was that, after inhaler technique training, active patients received a personalized inhaler technique label.

*NA* Not applicable.

^a^ Two active and one control ACC users did not attend last visit.

### Primary outcome-inhaler technique score after 3 months

At follow-up (*n* = 92), inhaler technique score had decreased in all groups from the post-education score of 9/9, but there was significantly less decline in active than control groups (Fig. 3a,b). The overall mean difference in score between randomization groups at 3 months was −1.29 (95% confidence interval (CI) −1.94, −0.64, *p* < 0.001). The mean difference in scores between active and control groups for ACC was −1.04 (−1.92, −0.16, *P* = 0.022) and for TH, −1.61 (−2.63, −0.59, *P* = 0.003) (Fig. 3a,b). However, scores at 3 months were still significantly higher than baseline pre-education scores for both active and control groups (Fig. 3a,b).

Multiple linear regression modeling indicated that randomization group was the only variable significantly associated with inhaler technique score at 3 months (*R* ^2^ = 0.184, *P* = 0.007, Table 3); baseline score (pre-education) and device type (ACC or TH) were not significant.

**Table 3:** Summary of the regression model obtained for the dependent variable, inhaler technique score at the 3 month assessment (*n* = 92)

Variable	Beta	*t*	*P* value
Type of inhaler (ACC and TH)	0.057	0.507	0.613
Randomization group (active or control)	−0.356	−3.568	**0.001**
Age	−0.102	−0.951	0.344
Baseline inhaler technique score	0.188	1.690	0.095
Gender	−0.068	−0.666	0.517
Smoking status	−0.032	−0.313	0.755

This table shows the output from a multivariable regression analysis in which inhaler technique score at 3 months was the dependent variable. “Beta” is the standardized regression coefficient. The overall fit of the model was *R* ^2^ = 0.184, *P* = 0.007.

### Proportion of participants with correct technique

For ACC, significantly more active patients still had correct technique at 3 months compared with control patients (62 vs. 12%, *p* < 0.001, Table 2). There was no significant difference for ACC active vs. control patients in the proportion with correct technique for the three ‘essential’ ACC steps.

For TH, significantly more active patients still had correct technique at 3 months compared with control patients (74 vs. 11%, *p* < 0.001), and significantly more active than control patients had correct technique for the four ‘essential’ TH steps (83 vs. 17%, *p* < 0.001).

### Asthma outcomes

**Fig. 4:**
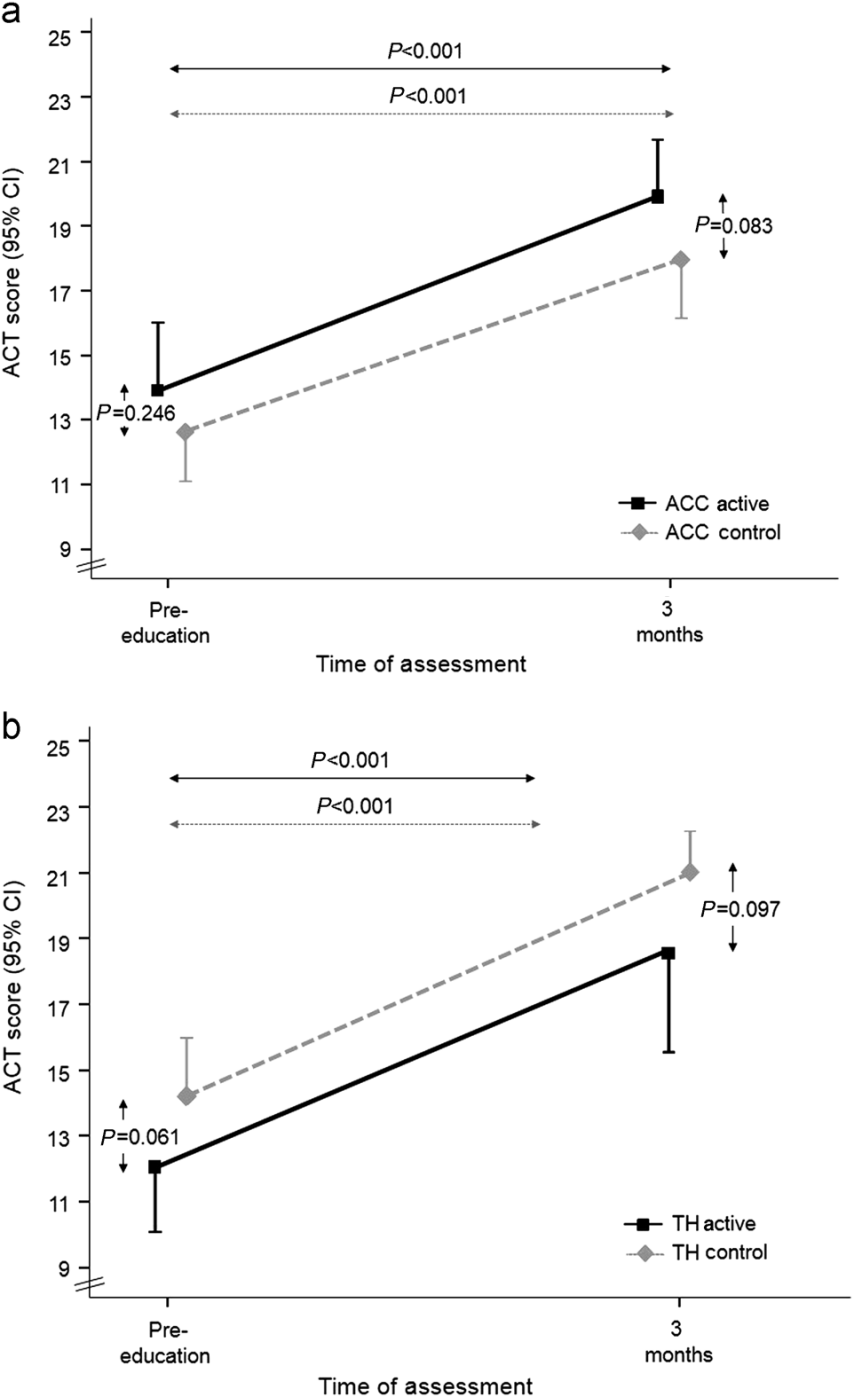
Asthma symptom control, assessed by ACT (Range 5–25, higher score is better symptom control). **a** Patients using the ACC in the active (*n* = 28) and control (*n* = 26) groups. **b** Patients using the TH in the active (*n* = 23) and control (*n* = 18) groups

For both randomization groups, asthma symptom control improved significantly by 3 months, with a mean increase in ACT score of 6.18 (SD 5.09) for active patients and 6.16 (SD 4.76) for control patients (mean difference between randomization groups 0.02 (−2.03, 2.07)). At the 3-month assessment, no significant difference in ACT score was found between active and control groups for either ACC (*P* = 0.083) or TH (*P* = 0.097) (Fig. 4a and 4b). No patients reported a change in their controller treatment since baseline.

Mean reliever use was significantly lower at follow-up for active vs. control patients 2.19 (SD 1.78) vs. 3.42 (1.83) puffs/day, *P* = 0.002 (Independent sample *t* test). Similar results were seen for patients using ACC (active 2.08 (1.83) vs. control 3.24 (2.13) puffs/day, *P* = 0.041) and TH (active 2.33 (1.74) vs. control 3.67 (1.33) puffs/day, *P* = 0.012).

## Discussion

### Main findings

This single-blind randomized controlled study confirmed that, in patients with asthma, poor inhaler technique can be corrected by a brief pharmacist-delivered Show and Tell educational intervention,^20^ and that, without further education, inhaler technique declines over the following 3 months. However, uniquely, the study showed that participants who had novel personalized inhaler technique labels placed on their inhalers after training had significantly better inhaler technique after 3 months, than those with initial training alone. With personalized labels highlighting their original errors, 67% of patients maintained correct technique at follow-up, compared with only 12% of those who received education alone. In this under-resourced asthma population with very poorly-controlled asthma at baseline, large improvements were seen in asthma symptom control in all groups at follow-up, consistent with the benefit of even a single brief intervention about inhaler technique; however, patients randomized to receive inhaler labels reported significantly less use of reliever medication by 3 months than those with education alone.

### Interpretation of findings in relation to previously published work

Several studies have shown that inhaler technique can be corrected by a variety of educational methods, but that it drops off after education. For example, de Blaquiere and colleagues showed that, of 62 patients with chronic lung disease with incorrect initial inhaler technique, 79% had correct technique after training but only 55% by 2 months later.^13^ Van der Palen and colleagues showed that, of 148 patients with COPD, the proportion with correct essential technique increased from 60% at baseline to 100% after training, but fell to 75% an average of 22 weeks later.^15^ Pothirat and colleagues reported that, of 103 elderly patients with COPD, 41% had correct technique at baseline, 100% after training, but only 51% at follow-up 1 month later.^16^

Several studies have examined factors contributing to baseline inhaler technique,^13, 16^ but Ovchinikova and colleagues examined factors contributing to short-term retention of inhaler technique. Amongst 127 patients with asthma (of whom 61% retained correct technique 1 month after education), baseline level of asthma symptom control, motivation to follow the correct steps when using the inhaler, and device type (DPI vs. pMDI) were independently associated with higher 1-month inhaler technique score.^14^ In the present study of DPIs, only randomization group (with/without labels) was significantly associated with retention of correct technique at 3 months.

We have previously shown that good inhaler technique was maintained over 3 months with a monthly pharmacist intervention, but that inhaler technique scores and the proportion of patients with correct technique decreased significantly over the following 3 months when no additional training was provided.^19^ In that study, novel personalized inhaler technique labels, conceived by IB, were used for the first time; however, since all patients received the labels, their independent effect could not be assessed. To our knowledge, similar labels have been used only in the study by Ovchinikova,^14^ again for all patients. The present study shows, for the first time, that placing personalized technique labels on the inhaler after education leads to better retention of correct inhaler technique over 3 months, compared with education alone.

Interval education alone may not be sufficient to change behavior in patients with chronic illness, and finding ways to optimize long-term self-management skills is vital.^21^ As the above studies show, it is not enough to deliver inhaler training only on a single occasion. Once a patient leaves the pharmacy, their main source of information about inhaler technique is the leaflet packaged with the inhaler. However, patients with asthma rarely read these, and often throw them away.^22^ Some studies have used take-home materials, such as written instructions and video instructions.^23, 24^ Van der Palen and colleagues provided all patients with a copy of the inhaler technique checklist marked with their errors.^15^ However, patients must remember and choose to use such supplementary material for it to provide any benefit. By contrast, the inhaler technique label, being attached to the device itself, is seen every time the patient uses it.

Improving patients’ inhaler technique skills has been shown previously to improve asthma outcomes, including lung function.^19, 20, 25^ In the present study, asthma symptom control over the previous 4 weeks, assessed by ACT, improved markedly in all patients, with mean improvement more than twice the minimal important difference of 3.0,^26^ and with no difference between randomization groups. Contributory factors may include that the study was conducted in a low-resource country in which few patients have access to asthma education,^7^ and most patients had very poorly controlled asthma at baseline, giving substantial room for improvement following any educational intervention; improved symptom control may also have led to improved adherence. Given the higher inhaler technique scores and lower reliever use in the active group at 3 months, longer follow-up may have revealed a difference between groups in symptom control or exacerbations.

### Strengths and limitations of this study

Strengths of the study include rigorous checking of inhaler technique using published checklists^8^ which provide reproducible scores,^27^ the use of a brief ‘show-and-tell’ inhaler technique intervention that is feasible and effective,^12, 19, 28^ use of a validated measure of asthma symptom control, and assessment of the primary outcome measure (change in inhaler technique score over 3 months) by a researcher blinded to the participants’ randomization group, with confirmation of inter-rater reliability prior to study start. Limitations include the large study effect on asthma control, and the withdrawal of four participants from one group prior to inhaler assessment, for differing reasons. Inhaler technique was extremely poor at baseline, but this is consistent with our findings using similar methodology in other studies.^7, 12, 20, 28^

### Implications for future research, policy and practice

The findings of this study confirm the high prevalence of poor inhaler technique with DPIs amongst asthma patients, and that inhaler technique assessment and education, although highly effective for improving both inhaler skills and asthma control, cannot simply be provided on a single occasion. Recent asthma guidelines emphasize the importance of checking and correcting inhaler technique at every opportunity.^1^ A recent review stressed the importance of research to identify optimal methods for translating evidence based medicine into implementation in everyday clinical practice.^29^ In our previous study,^14^ inhaler technique education was provided by pharmacists monthly for 3 months, whereas in this study, there was a 3 month interval after initial training, a more feasible model for clinical practice. Inhaler technique labels can thus increase the efficiency of inhaler education, by decreasing the need for frequent health professional review.

The novel inhaler labels investigated in this study are important tools for personalizing inhaler education, by highlighting each patient’s incorrect technique step/s. The labels present a simple visual cue that can provide daily customized instruction for patients, and act as a continuous reminder of correct technique.^19^ The findings should be confirmed in a range of health systems. The content of the labels is obviously constrained by the space available on the inhaler, and by patient literacy and visual acuity; electronic inhaler devices can already deliver audiovisual reminders for missed doses,^30^ and future research could investigate the potential for inhalers to talk the patient through the steps needed for good medication delivery.

Interestingly, at follow-up, most patients made different errors to the ones initially highlighted on their labels; the highlighted steps could possibly have directed the patient to focus on them to the detriment of the other steps. However, despite this, total inhaler scores were higher and correct technique was more likely to be maintained by patients randomised to receive labels. Since the label would be seen every time the inhaler was used, it is possible that the highlighting on its own could have contributed to improved technique, regardless of the steps highlighted, by maintaining awareness of the importance of inhaler technique. Further research in this area of patient education would be valuable.

Use of inhaler technique labels also provides an effective method for maintaining the inhaler skills of health professionals themselves. Many health professionals are not able to demonstrate correct inhaler technique,^31^ and, like patients, their skills fall off after training.^32–34^ However, we have previously shown that community pharmacists engaged in regularly checking their clients’ inhaler technique with checklists and inhaler labels maintained their own technique 2 years after a brief workshop.^6^ The labels can act as a quick and feasible tool, reminding pharmacists of the importance of educating and re-educating patients on correct inhaler technique.

## Conclusion

Poor inhaler technique is a major problem contributing to the burden and risk of asthma, and maintenance of correct technique requires time and resources for repeated education. This study shows that retention of correct inhaler technique with DPIs can be enhanced by attaching a personalized label to the inhaler, highlighting the patient’s own technique errors. The labels represent an inexpensive, feasible, scalable intervention that increases the clinical efficiency of inhaler training, and has the potential to extend the resulting improvement in asthma outcomes.

## Methods

This 3-month single-blind randomized parallel-group active-controlled study was conducted in 2010. Consecutive patients with asthma visiting respiratory clinics at two large hospitals in Amman, Jordan, and using controller medication by ACC or TH were approached by the researcher for participation. Ethics approval was obtained from the Jordanian Ministry of Health and from the hospitals at which the study was conducted, and patients gave written informed consent. Patients were informed that the study was about asthma management, with no mention of inhaler technique. Inclusion criteria were: age ≥14 years, doctor diagnosis of asthma, currently using a controller medication (inhaled corticosteroid (ICS) with or without long-acting β_2_-agonist) via TH or ACC, and having been on the same medication and dose for ≥1 month prior to study enrollment. Patients were excluded if they did not self-administer their medication, were not able to return for all visits, were involved in another clinical study, or did not speak or understand Arabic (the official language for medical care in Jordan).

The study was preceded by a 1-month pilot study at the same hospitals to assess feasibility, identify and address barriers, and assess clarity and readability of the questionnaires.

### Baseline assessments

At baseline, data about demographics, asthma medications, age at diagnosis of asthma and reliever use in the previous month were collected. Asthma symptom control was assessed using a published Arabic translation of the 5-item asthma control test (ACT).^35^

After randomization (as below), patients’ technique with their controller device (TH or ACC) was assessed by a trained researcher using placebo inhalers provided by AstraZeneca Pharmaceuticals (Wilmington, Delaware; Amman, Jordan) and GlaxoSmithKline (Philadelphia, Pennsylvania; Amman, Jordan), and validated inhaler technique checklists (see Supplementary online appendix),^28, 36^ translated into Arabic. The checklist for each device consisted of 9 steps (potential score 0–9). For the TH, four steps were classified as “essential” (steps without which little or no medication would reach the airway), and for the ACC, three steps were classified as essential (see Supplementary online appendix).^36, 37^

### Study interventions

According to the protocol, after patients completed the baseline questionnaires, the researcher randomized them to active or control interventions using a computerized list, with stratification by baseline level of asthma control (ACT ≤19 or >19)^35^ and by type of inhaler; the focus on inhaler technique was concealed until after baseline questionnaires. After randomization, inhaler technique was assessed (as above) and then optimized for both active and control group patients, using identical educational methods based on our previous published research.^20^ In this specialized “Show and Tell” inhaler technique counseling service, the researcher went through each step on the device-specific checklist with the patient in Arabic, to describe and demonstrate correct use. This cycle of assessment and counseling was repeated up to three times if necessary, until the patient demonstrated correct technique on all steps (score 9/9).^20^

For the active intervention group, the researcher then used a highlighter pen to identify all incorrect steps from the patient’s initial (pre-education) assessment on an “Inhaler Technique Label”, which was preprinted in Arabic with the relevant device checklist. The researcher attached the highlighted label to the patient’s controller inhaler (not the box), without covering any essential information (Fig. 1). Patients were provided with three extra highlighted labels to attach to new inhalers as they were purchased.

### Follow-up

Each month, the researcher made a brief (<1 minute) telephone call to all participants (active and control) to ask if they had purchased a new inhaler. Active participants who had purchased a new inhaler were reminded to attach a label to their new inhaler. Inhaler technique was not mentioned in the telephone calls for either group.

Three months after the baseline visit, a second trained researcher, blinded to randomization, assessed all participants on their inhaler technique, asthma control, controller and reliever use. The two researchers who assessed technique at baseline (pre and post-education) and at 3-month follow-up were trained by one investigator (IB). Inter-rater reliability, based on assessment of six non-study patients, showed a mean difference between assessment scores of 0.23 out of 9 (95% CI −0.09 to 0.54) (Bland and Altman analysis of difference).

After the final assessment, participants in both groups were re-trained in inhaler technique and received inhaler technique labels.

### Data analysis

The primary outcome was change in inhaler technique score between post-education and 3 months. Data were analyzed with SPSS 20 (Chicago, Illinois). Differences with *P* < 0.05 were considered statistically significant. The proportions of patients who performed correct technique (all steps correct) and correct essential technique (essential steps correct) were compared with Pearson’s *χ* ^2^ test. For continuous variables (inhaler technique scores, ACT scores), comparisons between groups were performed by Independent Sample *t* test, Wilcoxon signed-rank test, and Mann–Whitney *U*-test.

In order to determine predictors of inhaler technique improvement at 3 months, a multiple linear regression analysis was performed. The dependent variable was inhaler technique score at 3 months. Independent variables included inhaler type (TH or ACC), intervention group (active or control), baseline inhaler technique score, gender, age and smoking status.

## Electronic supplementary material

Supplementary Information
