# Supplementary material for: Effect of novel inhaler technique reminder labels on the retention of inhaler technique skills in asthma: a single-blind randomized controlled trial
Source: NPJ Prim Care Respir Med. 2017 Feb 9;27:9. doi: 10.1038/s41533-017-0011-4 (PMC5434787; doi:10.1038/s41533-017-0011-4)
Supplement: Supplementary file 1 — Supplementary Information [file 41533_2017_11_MOESM1_ESM.docx]

**Online Appendix**

Accuhaler [Diskus] Technique Checklist

| Step. Description/Action |
| --- |
| 1. Open Inhaler* |
| 2. Push lever back completely* |
| 3. Exhale to residual volume |
| 4. Exhale away from mouthpiece |
| 5. Place mouthpiece between teeth and lips |
| 6. Inhale forcefully and deeply* |
| 7. Hold breath for 5 seconds |
| 8. Exhale away from mouthpiece |
| 9. Close inhaler |

Turbuhaler Technique Checklist

| Step. Description/Action |
| --- |
| 1. Remove the cap from the Inhaler* |
| 2. Keep inhaler upright* |
| 3. Rotate grip until a click is heard* |
| 4. Exhale to residual volume |
| 5. Exhale away from mouthpiece |
| 6. Place mouthpiece between teeth and lips |
| 7. Inhale forcefully and deeply* |
| 8. Hold breath for 5 seconds† |
| 9. Exhale away from mouthpiece |

These checklists and essential steps are based on published evidence and manufacturers’ instructions.[^36^](#_ENREF_36) They were translated into Arabic for the present study.

* Essential step: if not performed correctly, little or no medication will reach the lung.

† Considered essential by van der Palen and colleagues.[^37^](#_ENREF_37)

‡ This step is not included in the product insert but appears in the Turbuhaler instructions on the Global Initiative for Asthma Web site,[^5^](#_ENREF_5) and in the checklist from van der Palen and collegues.[^37^](#_ENREF_37) Harmonization of instructions about breath-holding is recommended across all devices to avoid confusion for health professionals and patients.[^36^](#_ENREF_36)
